# Explainable Artificial Intelligence and Wearable Sensor-Based Gait Analysis to Identify Patients with Osteopenia and Sarcopenia in Daily Life

**DOI:** 10.3390/bios12030167

**Published:** 2022-03-07

**Authors:** Jeong-Kyun Kim, Myung-Nam Bae, Kangbok Lee, Jae-Chul Kim, Sang Gi Hong

**Affiliations:** 1 Department of Computer Software, University of Science and Technology, Daejeon 34113, Korea; kim.jk@etri.re.kr; 2 Intelligent Convergence Research Laboratory, Electronics and Telecommunications Research Institute, Daejeon 34129, Korea; mnbae@etri.re.kr (M.-N.B.); kblee@etri.re.kr (K.L.); kimjc@etri.re.kr (J.-C.K.)

**Keywords:** osteopenia, sarcopenia, XAI, SHAP, IMU, gait analysis

## Abstract

Osteopenia and sarcopenia can cause various senile diseases and are key factors related to the quality of life in old age. There is need for portable tools and methods that can analyze osteopenia and sarcopenia risks during daily life, rather than requiring a specialized hospital setting. Gait is a suitable indicator of musculoskeletal diseases; therefore, we analyzed the gait signal obtained from an inertial-sensor-based wearable gait device as a tool to manage bone loss and muscle loss in daily life. To analyze the inertial-sensor-based gait, the inertial signal was classified into seven gait phases, and descriptive statistical parameters were obtained for each gait phase. Subsequently, explainable artificial intelligence was utilized to analyze the contribution and importance of descriptive statistical parameters on osteopenia and sarcopenia. It was found that XGBoost yielded a high accuracy of 88.69% for osteopenia, whereas the random forest approach showed a high accuracy of 93.75% for sarcopenia. Transfer learning with a ResNet backbone exhibited appropriate performance but showed lower accuracy than the descriptive statistical parameter-based identification result. The proposed gait analysis method confirmed high classification accuracy and the statistical significance of gait factors that can be used for osteopenia and sarcopenia management.

## 1. Introduction

Osteopenia and sarcopenia can cause various senile disorders and are key factors related to the quality of life in old age [1,2,3]. Gait is a suitable indicator of musculo-skeletal diseases [4]. With the miniaturization of sensors and the development of intelligent monitoring technology, interest in wearable-sensor-based daily health management solutions is increasing [4,5]. Therefore, portable tools and methods that can analyze osteopenia and sarcopenia risks in our daily lives, rather than requiring a specialized hospital setting, can be considered.

Musculoskeletal disorders are increasingly being recognized as conditions that are associated with significant morbidity, mortality, and healthcare costs [1,2]. Osteopenia is a cause of fracture and increases the risk of complications, in addition to pain caused by fractures. Osteoporotic fractures generate costs that reach USD 25 billion, and sarcopenia generates costs of approximately USD 18 billion [2,6]. Patients with sarcopenia have a slow gait, reduced muscular endurance, face difficulty in daily living, and frequently need help from others. Osteoporosis, falls, and fractures can occur easily, whereas the blood and hormonal buffering action of the muscle are moderated, reducing the basal metabolic rate, making chronic diseases unmanageable, and increasing the likelihood of aggravating diabetes and cardiovascular disease [3]. 

Osteoporosis is defined by the World Health Organization (WHO) as a medical condition in which the bone mineral density (BMD) is less than −2.5 standard deviation (SD) below the mean level for young adults, and for osteopenia it is between −2.5 and −1.0 [7]. Sarcopenia is defined by the European Working Group on Sarcopenia in Older People (EWGSOP) as the presence of low muscle mass, reduced muscle strength, and physical performance [8]. BMD and muscle mass are diagnosed via dual-energy X-ray absorptiometry (DEXA) [1], although cannot be measured without expert assistance. Therefore, a system that can easily manage musculoskeletal diseases in daily life is required. 

Human gait involves interactions between the musculoskeletal system and the nervous system. Thus, gait analysis is effective in identifying neuromusculoskeletal disorders such as Parkinson’s diseases (PD) [9,10], fall risk [11,12], total hip arthroplasty (THA) [5,13], and sarcopenia [4]. Traditionally, cameras and force plates have been used as clinical gait assessment tools; however, these tools are used only in large institutions such as university hospitals and are difficult to apply in daily life or complex environments because of their high cost and large space requirements [14]. Given the recent miniaturization and increased accuracy of sensor technology, inertial measurement units (IMU) are increasingly being used for gait analysis [4]. 

Gait analysis methods include statistical comparisons of gait parameters obtained from control and target groups and a method of analysis of the classification results of the groups using machine learning. In the analysis of gait for osteopenia, osteoporosis, sarcopenia, and osteosarcopenia conducted by Intriago [2], the slowest walking speed was observed in osteosarcopenia: 0.9 m/s in osteopenia–osteoporosis, 0.893 in sarcopenia, and 0.7 in osteosarcopenia. Choi [15] investigated the correlation between kinetic gait parameters and femoral BMD of the femoral neck, trochanter, shaft, and total proximal femur. The highest correlation (r = 0.153, *p* = 0.014) was observed between the walking speed and femoral neck BMD among the older female participants. ElDeeb [16] aimed to investigate the gait characteristics of postmenopausal women with low BMD (n = 17) and to determine the predictive parameters of BMD. When the normal BMD group and women with low BMD were compared, the ankle joint showed less push-off (*p* = 0.000), which seemed to be used to obtain gait stability. Sung [17] divided 77 older participants (n = 48 female + 29 male) into normal BMD and low BMD groups using DEXA. The spatial–temporal gait parameters (speed, stride length, and support times) of both groups were subsequently investigated. The support times included those of the initial double support, single support, and terminal double support in the stance phase. The support time was confirmed to have a high ratio of the main foot (the foot mainly used), the stride length was found to be longer on the main foot side than on the other side, and the stride length was positively associated with the single support time on the dominant limb. 

Although there are many studies on gait speed for osteopenia and sarcopenia, only a few studies have analyzed gait parameters such as PD, fall risk, and THA. Recently, explainable artificial intelligence (XAI) has received considerable attention as a method to analyze the importance and contribution of parameters. XAI presents predictive results for machine learning in a human-understandable form [18]. It is primarily used to enhance the reliability of machine learning results. Low machine learning accuracy results in the misinterpretation of XAI. The XAI technique detects feature importance and explains the influence of features on model decisions [19]. Therefore, for the management of osteopenia and sarcopenia in daily life, this study proposes an algorithm for detecting gait parameters and identifying patients based on inertia signals and interpreting the results using XAI.

## 2. Related Studies

This section describes research related to gait parameter detection techniques, wearable-sensor-based patient identification, and XAI. This information should help with the understanding of the proposed wearable-based gait analysis method for the management of osteopenia and sarcopenia in daily life. This study proposes an algorithm for detecting gait parameters based on inertial signals, identifying patients, and interpreting the results using XAI for gait analysis.

### 2.1. Gait Parameter

IMU-based gait analysis is used to identify PD, fall risk, THA, and sarcopenia. Gait parameters for analysis based on IMU include spatial–temporal parameters (e.g., step length, stance phase, swing phase, single support, double support, step time, cadence, and speed), kinematic parameters (the rotational angles of the sagittal, coronal, and transverse plane of the pelvis, hip, knee, and ankle), and descriptive statistical parameters (such as the maximum, mean, and standard deviation) of inertial signals for each gait phase. The results of gait analysis using spatial–temporal parameters can be compared with the results of other gait analysis tools such as cameras and force plates. However, the disadvantage is that the motion information acquired by the inertial sensor is reduced, resulting in a low classification result [4]. 

Gait events and phases are detected to extract the gait parameters. Taborri [20] classified gait into two to eight phases. Whittle [21] classified gait into seven phases, and this is the most widely used classification method. One stride is from a heel strike to the next heel strike. Gait is broadly classified into a stance phase from heel strike (HS) to toe off (TO) and a swing phase from TO to the next HS. The stance phase is classified into the loading response, mid stance, terminal stance, and pre swing phases, and the swing phase is classified into the initial swing, mid swing, and terminal swing phases. The spatial–temporal parameters can be obtained by extracting the HS, TO, opposite HS, opposite TO, and walking distance. HS and TO are detected by the time and frequency signal processing of the inertial signal. Kim [22] obtained HS and TO with high accuracy within 0.03 s through time–frequency analysis. 

The inertial-sensor-based distance measurement algorithm is widely used as the basis for the distance measurement algorithm in indoor navigation research, and it is difficult to accurately measure the distance using only the inertial sensor [23,24]. Kinematic parameters are obtained by attaching inertial sensors to locations such as the pelvis, hip, knee, and ankle, but they are difficult to use in daily life due to the number of sensors required. Descriptive statistics and frequency analysis have been employed to analyze the signal obtained from the inertial sensor. 

### 2.2. Identifying Patients Based on Inertial Signals

IMU-based gait analysis is used to identify PD, fall risk, THA, and sarcopenia. With gait-parameter-based disease identification, Caramia [9] classified PD using linear discriminant analysis (LDA), naïve Bayes (NB), k-nearest neighbor (k-NN), support vector machine (SVM), SVM radial basis function (RBF), decision tree (DT), and the majority of votes. The performance of the machine learning technique—SVM with a nonlinear kernel—was the best. Eskofier [10] analyzed the gait of PD using descriptive statistical parameters such as the energy maximum, minimum, mean, variance, skewness, and kurtosis of the signal measured by the inertial sensor and the fast Fourier transform, a frequency analysis method. Howcroft [11] predicted the risk of falls using accelerometer data and used temporal (cadence and stride time) and descriptive statistics (maximum, mean, and SD of acceleration). NB, SVM, and neural network (NN) were used as classification methods, and the best single-sensor model was NN. An advantage of deep learning is that it can detect features within the algorithm from a raw signal, although Tunca [12] achieved higher accuracy in long short-term memory (LSTM) when certain parameters (e.g., speed, stride length, cycle time, stance time, swing time, clearance, stance ratio, and cadence) were used as the inputs, compared with raw signals.

Teufl [5] classified THA patients using stride length, stride time, cadence, speed, hip, and pelvis range of motion (ROM) as features of the SVM, and obtained an accuracy of 97%. Dindorf [13] used local interpretable model-agnostic explanations (LIME) to understand the features for identifying THA, and found that the sagittal movement of the hip, knee, and pelvis, as well as transversal movement of the ankle, were particularly important for this specific classification task. Kim [4] obtained feature importance using the Shapley additive explanations (SHAP) approach for spatial–temporal parameters and descriptive statistical parameters detected from signals measured by inertial sensors on both feet of ten sarcopenia and control participants. Twenty descriptive statistical parameters of high importance were used as inputs to classification models such as SVM, RF, and multi –layer perceptron (MLP); the highest accuracy (95%) was achieved using the SVM model, as shown in Table 1.

### 2.3. Explainable Artificial Intelligence 

XAI is a method that allows humans to understand the basis of decisions made by artificial intelligence models [25]. It is primarily used to enhance the reliability of machine learning results. Low machine learning accuracy results in a misinterpretation of XAI. The XAI technique detects feature importance and explains the influence of features on model decisions [19]. LIME and SHAP are often used to explain existing handcraft feature-based classification algorithms, and layer-wise relevance propagation (LRP) and class activation mapping (CAM) are used as algorithms to interpret deep learning.

LIME is effective for tabular data, text, and images. However, it is difficult to set the kernel width in tabular data, and different results are obtained during repeated execution because the sampling process is performed randomly. SHAP was proposed to consider the dependency between features. When the dependence between features is high, the SHAP feature importance is judged to be better than the permutation importance [19]. SHAP is based on Shaply values from game theory. The main advantages of the SHAP method are local explanations and consistency in the global model structure. SHAP is used in many machine learning models as a model-agnostic method. 

LRP outputs a heatmap to the input image by tracing back the results of the deep learning model. Unlike LIME and SHAP, which interpret the model using the sensitivity analysis technique, LRP [25] is a mixture of relevance propagation and decomposition. Relevance propagation is a method for calculating the relevance of the contribution of the hidden layer to the output after the decomposition process. CAM visualizes model decisions by computing a weighted linear summation of the last convolutional feature map. CAM is limited to model architectures where the model must consist of one fully connected (FC) layer with global average pooling (GAP) [26]. Grad-CAM describes models without constraints on the model architecture. Gradient-based CAM methods share the problem of shattered gradients, causing noise saliency maps in the intermediate layer. An LRP-based Relevance-CAM has been proposed to solve the gradient problem [27].

## 3. Methods

**Figure 1:**
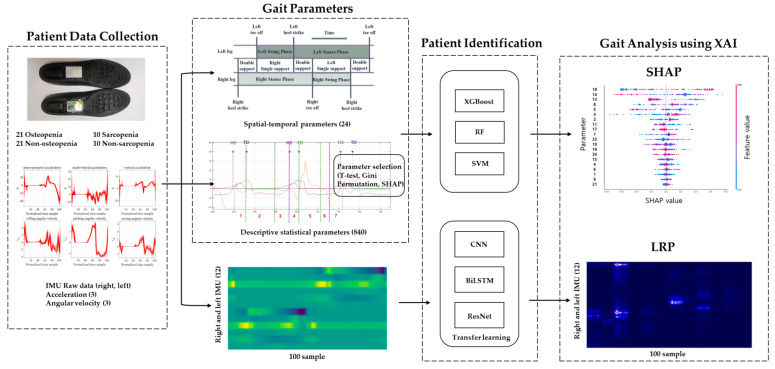
Gait analysis flowchart.

To analyze the gait of the osteopenia and sarcopenia groups, the patients were identified using machine learning, and the machine learning model was interpreted using XAI. The inertial sensor signals and spatial–temporal and descriptive statistical parameters detected in the proposed algorithm were used as machine learning inputs. By analyzing the model that obtained high-accuracy identification results, the inertial signal and gait parameters of the osteopenia and sarcopenia groups were analyzed. The flowchart of patient identification and gait analysis for osteopenia and sarcopenia is shown in Figure 1.

### 3.1. Patient Data Collection 

Gait signals of 42 women over 65 years of age were obtained to analyze the gait characteristics for osteopenia and sarcopenia. Among the 42 subjects, there were 21 patients with osteopenia and 21 patients without osteopenia. The BMD obtained by measuring DEXA was compared with that of a healthy young person: when the T-score was −1 SD or higher, the data were assigned to the control group; when it was lower than −1, the data were assigned to the osteopenia group. Additionally, 10 sarcopenia and 10 non-sarcopenia patients were selected among the 42 subjects. Sarcopenia was diagnosed using the skeletal muscle mass index (SMI, appendicular skeletal muscle mass in kg/height in m^2^) that was less than 5.4 kg/m^2^ (as obtained through DEXA), whereas the grasp strength was less than 18 kg. The group without sarcopenia included participants with SMI of 5.5 or more and a grasp strength of 19 kg or more. Relevant statistics, including age, height, weight, foot size, Mini-Mental State Examination (MMSE) [28], the Mores Fall Scale (MFS) [29], SARC-F questionnaire [30], Berg Balance Scale (BBS) [31] and Timed Up and Go (TUG) scores [32], grasp power, T-score for DEXA, and SMI, are shown in Table 2. 

The limitations of this study were that the physiological and psychological variables of the participants could not be controlled, the age range of the participants could not be expanded, the study was conducted on women only, and the treadmill gait experiment with fall risk factors for older adults was excluded, and only the preferred speed through walking on flat ground was measured.

**Figure 2:**
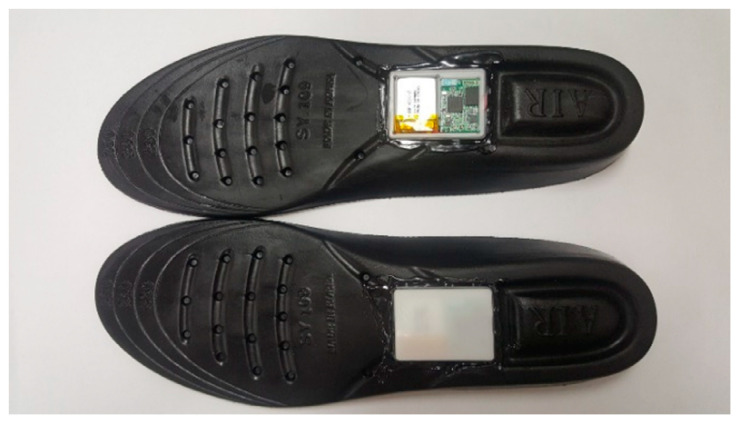
Sensor attachments to the insoles.

All participants wore the same sneaker model and walked the 27 m corridor four times in a straight line. The gait data were acquired from the right and left insoles using IMU, as shown in Figure 2. The IMU settings included an acceleration sensitivity of 8G, a gyro sensitivity of 1000°/s, and a sampling frequency of 100 Hz [4]. 

Additionally, 20 participants measured 9 m gait simultaneously with the clinical standard system and the proposed inertial system to verify the proposed device and algorithm. The clinical system consisted of ten cameras (Vicon, Oxford Metrics, Oxford, UK) and four force plates (Advanced Mechanical Technology, MA, USA). Data analysis was performed using the Vicon Polygon 3.5.2. Ethics approval was obtained from the Chungnam National University Hospital Institutional Review Board before conducting this study (File No: CNUH 2019-06-042).

### 3.2. Gait Signals and Parameters 

**Figure 3:**
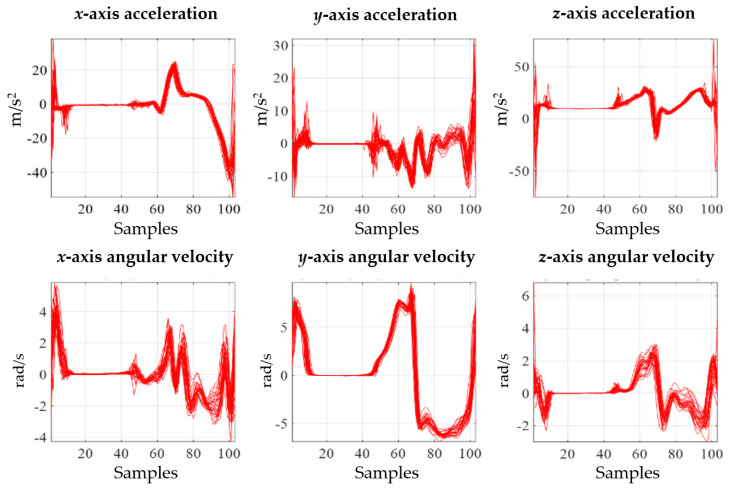
Acceleration and angular velocity signals.

Gait is a motion in which both feet alternately repeat the stance and swing phases, and the event points of gait that separate the stance and swing phases are called HS and TO. HS is at the start of the stance phase, and TO is at the start of the swing phase. The gait data obtained from the IMU sensor were 6-axis signals that included the *xyz*-axis acceleration and angular velocity signal. When the measured sensor data were separated based on HS and normalized to 100 samples, they exhibited periodic characteristics, as shown in Figure 3. The characteristics of the gait signal differ from person to person, and for IMU gait analysis, the spatial–temporal parameter was detected from the gait signal, and the gait signals were expressed as descriptive statistical parameters and analyzed. Additionally, patients were classified using raw data as inputs for deep learning without detecting the parameters; then, the gait signals were analyzed by interpreting the deep learning results.

The spatial–temporal parameters were extracted from the inertial signals using the proposed algorithm [4]. Twenty-four spatial–temporal parameters were detected: stance phase time right, stance phase time left, swing phase time right, swing phase time left, stance phase percent right, stance phase percent left, double support first phase time right, double support first phase time left, double support second phase time right, double support second phase time left, single support phase time right, single support phase time left, double support first phase percent right, double support first phase percent left, double support second phase percent right, double support second phase percent left, single support phase percent right, single support phase percent left, stride length right, stride length left, stance phase time SI, swing phase time SI, stance phase percent SI, and cadence. The definitions are summarized in Table 3.

After detecting HS and TO, the opposite HS, opposite TO, cadence, stance phase (time), swing phase (time), single support phase (time), and double support phase (time) could be obtained by arithmetic calculations. Secondary parameters, such as balance of difference between the right and left foot, were also collected through comparative analysis of both feet. Stride was detected through a distance estimation algorithm based on zero-velocity detection (zero-velocity update) using an extended Kalman filter [23,24]. 

To obtain descriptive statistical parameters, the six-axis gait signal was classified into seven phases, as proposed by Whittle. The detection of HS, TO, heel rise (HR), feet adjacent (FA), and tibia vertical (TV) is required to classify seven phases; it was detected using the method proposed in a previous study [4]. Ten descriptive statistical parameters were obtained from signals classified into seven phases, and the descriptive statistical parameters were max., min., SD, AbSum, root-mean-square (RMS), kurtosis, skewness, MMgr, DMM, and Mdif. A total of 840 descriptive statistical parameters (both feet (2) × sensor signal (6) × gait phase (7) × (10 parameters)) were detected.

### 3.3. Patient Identification

To identify patients using the inertial gait signal and the proposed gait parameters, osteopenia and sarcopenia groups were classified through various models such as RF, XGBoost (Extreme Gradient Boosting), SVM, and deep learning models.

RF is a decision tree ensemble classifier that combines multiple single classifiers to obtain the result of each classification model either through majority vote or weighted average [33]. RF lowers the risk of overfitting by using some data and features from the training data. XGBoost is a decision tree ensemble model and improves the performance of the gradient boosting machine in terms of speed. Boosting models increase accuracy by iteratively updating the parameters of the previous classifier to reduce the slope of the loss function, thereby generating a robust classifier [33]. SVM is a binary classifier that aims to determine the optimal separation hyperplane that maximizes the margin between two classes. Kernel functions are used to map data to a higher-dimensional space; thus, an SVM can compute nonlinear decision boundaries [4].

The representative deep-learning-based models were convolutional neural network (CNN) and LSTM. A CNN is composed of one or more convolutional, pooling, FC, and dense layers. CNNs exhibit high performance in detecting and classifying features in images. Unlike LSTM, which only has forward hidden layers, BiLSTM has both forward and backward hidden layers. Therefore, it learns both before and after information and demonstrates high performance in time-series data. As a CNN backbone, ResNet exhibits excellent classification accuracy [34]. ResNet uses skip connections (or short connections) to pass the input from the previous layer to the next layer. This skip connection solves the gradient loss/burst problem, enabling deep neural networks. ResNet uses 18, 34, 50, 101, and 152 layers depending on the depth, and there are structural differences in approximately 50 layers. In particular, ResNet is a popular architecture despite the existence of other models that have improved performance in various fields. Moreover, it is a representative CNN architecture for which many supporting materials are available [34,35].

Transfer learning is applied as a solution to address the difficulty of training a model based on small datasets. In transfer learning, data similar to the target data are learned in advance and a specific layer is frozen, such that only the layer which is not frozen when learning the target data is learned [36]. Specific data characteristics can be overfitted, because patient identification is a binary classification. Therefore, person identification is pre-trained because high-resolution features can be detected by comparing and analyzing the gaits of multiple people.

### 3.4. Gait Analysis

The gait signals and parameters were analyzed using statistical methods and XAI techniques that interpret machine learning results. The independent t-test was used as a statistical method to compare the spatial–temporal parameters and descriptive statistical parameters. To improve the reliability of the machine-learning-based analysis method, a higher accuracy should first be obtained. Therefore, the osteopenia and sarcopenia groups were classified through various models, such as RF, XGBoost, SVM, and deep learning models. The CNN and LSTM models were used as the deep learning models.

Spatial–temporal parameters and 100 descriptive statistical parameters with low *p*-values of the t-test were used as inputs for RF, XGBoost, and SVM. The following RF parameters were used: number of trees = 50, max_depth = 30, and number of features = square root of the gait parameters. The XGBoost parameters were booster = gbtree, objective = binary:logistic, eta = 0.018, max_depth = 15, gamma = 0.009, subsample = 0.98, and colsample_bytree = 0.86. SVM explored the linear and RBF kernels, and the parameters were gamma = 1.0 and C = 5.0.

The 12 axes of acceleration and angular velocity signals obtained from both feet were applied to the deep learning models. We proposed a low-layer-based CNN and BiLSTM model and applied ResNet50. As the input of the deep learning model, a stride based on HS was detected and normalized to 100 samples using spline interpolation because the signal was collected at 100 Hz [4]. ResNet50 is reduced in size by pooling as the layers progress. The ResNet50 backbone cannot be used with an input of shape 12, and removing pooling lowers the accuracy. Therefore, the input shape (36,100) was generated by amplifying the signal of 12 axes threefold because the input size of ResNet50 must be 12 or more, and the kernel size was 3. The layers of each model are shown in Table 4. The parameters of the deep learning model were as follows: learning rate = 0.0005, training epoch = 100, batch size = 16, loss = CrossEntropyLoss, optimizer = Adam, and activation function = Rectified Linear Unit.

XGBoost can calculate the built-in importance (Gini importance) and permutation importance using the learned model. Permutation importance measures the increase or decrease in prediction error compared with the original data when the feature data are transformed [19]. Permutation importance does not consider the correlation between features; therefore, SHAP was proposed as a method to consider the dependency between features. In particular, the SHAP feature importance is considered to be better than the permutation importance because gait parameters have a high dependence on the features. The Gini, permutation, and SHAP importance of the spatial–temporal and descriptive statistical parameters were calculated to obtain important parameters of osteopenia and sarcopenia, and the results of deep learning were analyzed using LRP, Grad-CAM, and Relevance-CAM.

## 4. Results

### 4.1. Patient Identification

The identification results of 21 osteopenia and 21 non-osteopenia subjects showed the highest accuracy in SVM when 24 spatial–temporal parameters were used as inputs, but showed an accuracy of less than 65%. The descriptive statistics parameter obtained the highest accuracy of 68.45% in XGBoost by using 100 parameters with a low *p*-value as an input, as a result of an independent t-test. For training and testing, 21 cross-validations were performed on 21 subjects, and the average was obtained. Using an inertial sensor as an input for deep learning, ResNet showed the highest accuracy among CNN, BiLSTM, and ResNet. The results of applying transfer learning to the ResNet model showed lower accuracy than when no transfer learning was applied. However, when performing transfer learning, it was shown that the accuracy increased when features were extracted, including the test subject. This implies that ResNet was pre-trained for human identification using the data of 42 patients, and the patient identification was cross-validated for 21 patients. The osteopenia group obtained the highest recognition result in the transfer learning ResNet. 

The identification results of 10 sarcopenia and 10 non-sarcopenia cases were over 70% accurate in terms of the spatial–temporal parameters, and the accuracy in case of sarcopenia was better than that in osteopenia. When the descriptive statistics parameter was used as the RF input, the highest accuracy was obtained, and the deep learning method of the inertial sensor input did not yield satisfactory identification results. Therefore, analysis of the results of XAI based on parameters is more reliable than the analysis of results based on deep learning. The patient identification results of machine learning are presented in Table 5 and Table 6.

### 4.2. Importance of Descriptive Statistical Parameter

The order of Gini, permutation, and SHAP importance was obtained for the descriptive statistical parameters of osteopenia and sarcopenia. When using highly important parameters such as RF, XGBoost, and SVM inputs, SHAP obtained the highest identification rate; however, when using the inner 20 important parameters as inputs, more identification results than the 100 descriptive statistical parameters were obtained. Table 7 and Table 8 showed the classification results as the number of parameters increased, and the average accuracy was obtained by performing 21 cross-validations for osteopenia and 10 cross-validations for sarcopenia. According to the result of each cross-validation, SHAP-based feature importance has different values. For example, osteopenia was trained with 40 datasets (20 osteopenia datasets and 20 non-osteopenia datasets) and tested with two datasets (1 osteopenia dataset and 1 non-osteopenia dataset) during 21 cross-validations. As a result of the training, the Shapley values were obtained based on the training data of 40 people, and the Shapley values were obtained in different orders. Table 9 shows the average results for 20 high-order Shapley values generated during 21 cross-validations. In osteopenia, the Shapley value is relatively high in the upper parameter and less than 0.1 from the 10th parameter. In sarcopenia, the difference in the Shapley value between the parameters is small. The parameter numbers of descriptive statistical parameters are shown in Table 10. The results of learning RF, XGBoost, and SVM with 20 parameters with high importance in Table 9 are shown in Table 11. Osteopenia obtained an accuracy of 88.69% in XGBoost using the top 4 parameters as inputs, and sarcopenia obtained an accuracy of 93.75% in RF using the top 18 parameters as inputs.

### 4.3. Gait Analysis

In the spatial–temporal parameters of the osteopenia group, the stance phase percentage decreased, double support percentage (time) decreased, and single support percentage increased. The sarcopenia group showed an increase in the value of the SI parameter compared with the non-sarcopenia group, implying that the difference between both feet was large. Except for the SI parameter, the *p*-value did not have a statistical significance of less than 0.001. Table 12 shows the mean and Shapley values of the spatial–temporal parameters; * indicates that the *p*-value is less than 0.025, and ** indicates that the *p*-value is less than 0.001.

From the result of the SHAP plot of osteopenia, as the value of the single support phase percent left (parameter 18) increased, the risk of osteopenia increased, as indicated by the positive SHAP value. As the value decreased, the risk also decreased, with the SHAP value being negative. When the single support phase percent left value increased, the risk increased linearly, and the osteopenia risk was low, at 39 or lower, and the risk increased at 42 or higher. A low double support first phase percent (parameter 13 and 14) increased the risk of osteopenia, with a decreased risk above 9 and an increased risk below 9. A low value of double support first phase time left (parameter 8) increased the risk, a high value decreased the risk of osteopenia, and a double support first phase time left lower than 0.075 led to an increase in risk. A low value of the stance phase percent right (parameter 5) increased the risk. 

**Figure 4:**
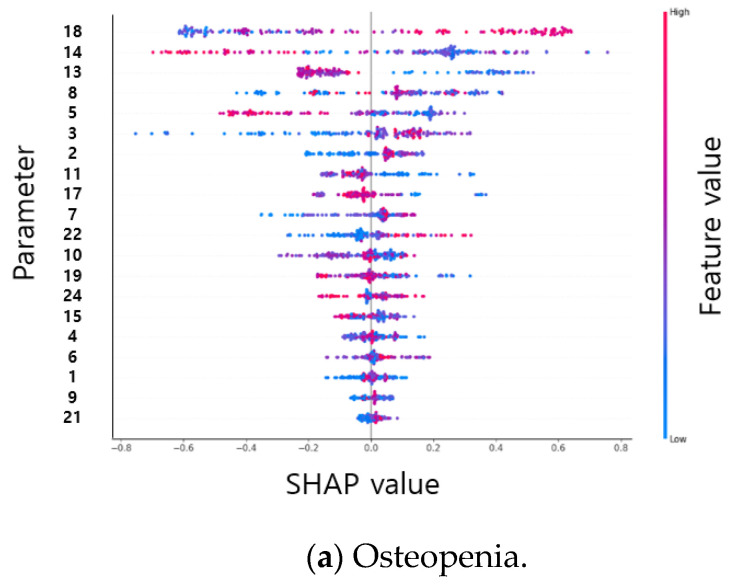

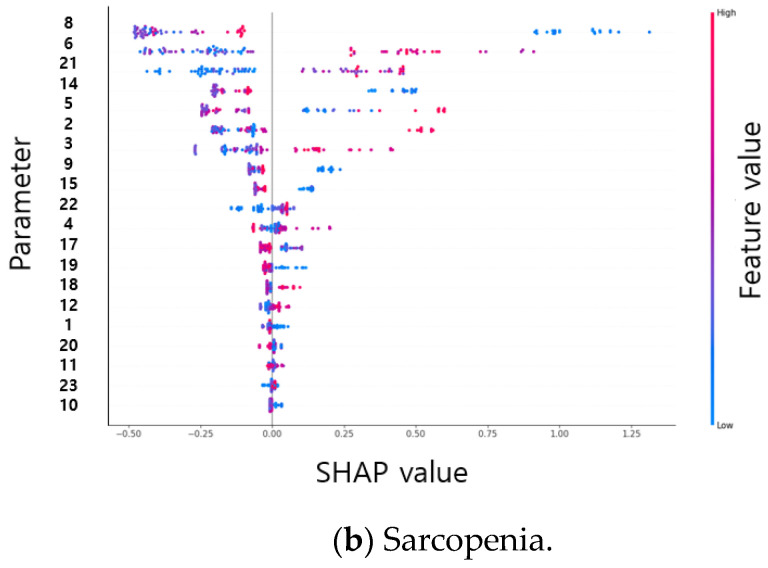
SHAP plots of the spatial–temporal parameters of osteopenia (**a**) and sarcopenia (**b**).

As a result of the SHAP plot of sarcopenia, the risk of sarcopenia increased when the double support first phase time left (parameter 8) had a very low value (less than 0.07). Stance phase percent left (parameter 6) increased the risk above 60 and decreased below 60, but did not show linearity. The risk increased when the value of the stance phase time SI (parameter 21) increased, and the risk was high at 0.35 or higher, although it was low at less than 0.35. SHAP plots of the spatial–temporal parameters of osteopenia and sarcopenia are shown in Figure 4.

**Figure 5:**
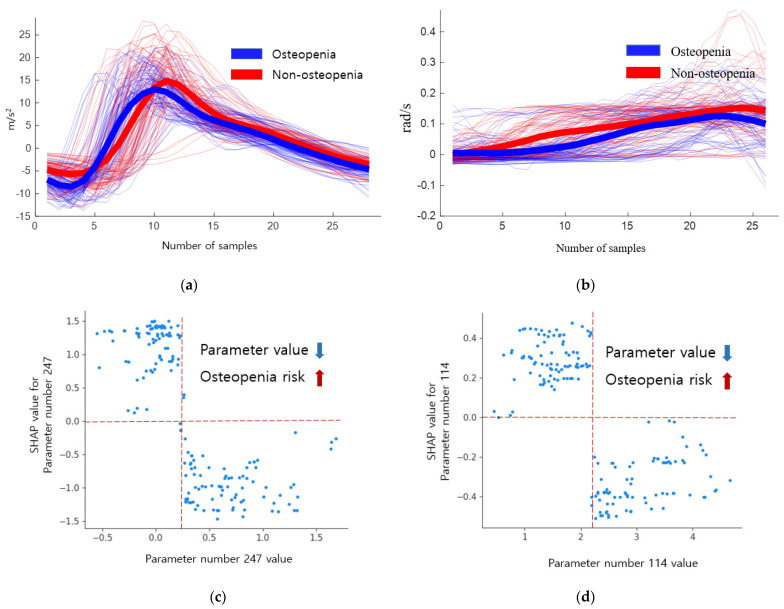
Inertial signals and SHAP dependence plots of descriptive statistical parameters 247 and 114 of osteopenia. (**a**) Inertial signal 247. (**b**) Inertial signal 114. (**c**) SHAP dependence plot 247. (**d**) SHAP dependence plot 114.

The parameter with the highest SHAP value within the descriptive statistical parameters of osteopenia is the skewness of the *x*-axis of the accelerometer in the initial swing phase (parameter number 247). Initial swing refers to the FA after TO. When the skewness is negative, the probability density function has a long tail on the left side, and the data, including the median, are more distributed on the right side. When the skewness is positive, there is a long tail on the right side of the probability density function, indicating that the data are more distributed on the left side. Skewness has a positive value when the mean is smaller than the median, negative when the mean is larger, and has a larger value as the difference between the median and the mean becomes larger. When there is a negative value, the right part of Figure 5a has a long tail, but the average decreases and the skewness value becomes small or negative. 

The inertial signals and SHAP dependence plots of the descriptive statistical parameters 247 and 114 of osteopenia cases are shown in Figure 5. The blue signal represents osteopenia, and the red signal represents non-osteopenia. A skewness of 0.5 or higher shows a low risk of osteopenia, whereas a negative value shows an increased risk. The absolute sum of gyro z values in the mid stance (parameter number 114) decreased in osteopenia. This implies that there is no rotation of the z-axis in the mid stance. When the absolute sum of values was 2.6 or higher, the risk decreased, and when the absolute sum of values was 1.89 or lower, the risk increased, as shown in Table 13. 

**Figure 6:**
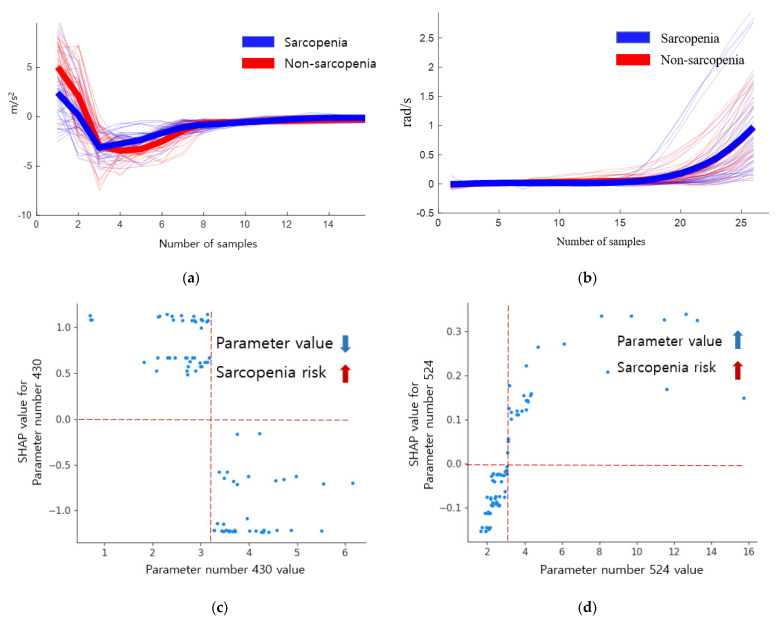
Inertial signals and SHAP dependence plots of descriptive statistical parameters 430 and 524 of sarcopenia. (**a**) Inertial signal 430. (**b**) Inertial signal 524. (**c**) SHAP dependence plot 430. (**d**) SHAP dependence plot 524.

The inertial signals and SHAP dependence plots of the descriptive statistical parameters 430 and 524 of sarcopenia are shown in Figure 6. Here, the blue signal represents sarcopenia, whereas the red signal represents non-sarcopenia. The maximum difference between two successive values of accelerometer x in the loading response (parameter number 430) was lower in the sarcopenia group than in the non-sarcopenia group. When the maximum value was less than 2.74, the risk of sarcopenia increased; however, when the maximum value was 3.79 or more, the risk of sarcopenia decreased. In the sarcopenia group, the change in the acceleration was smooth. The absolute sum of gyro y values in the mid stance (parameter number 524) increased in the sarcopenia group. As the absolute sum value increased, the risk of sarcopenia increased.

**Figure 7:**
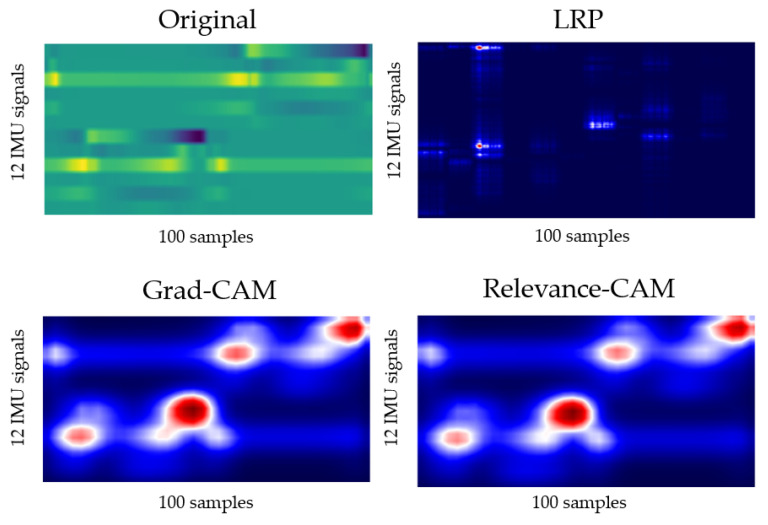
Layer2 result of applying LRP, Grad-CAM, and Relevance-CAM to ResNet50.

**Figure 8:**
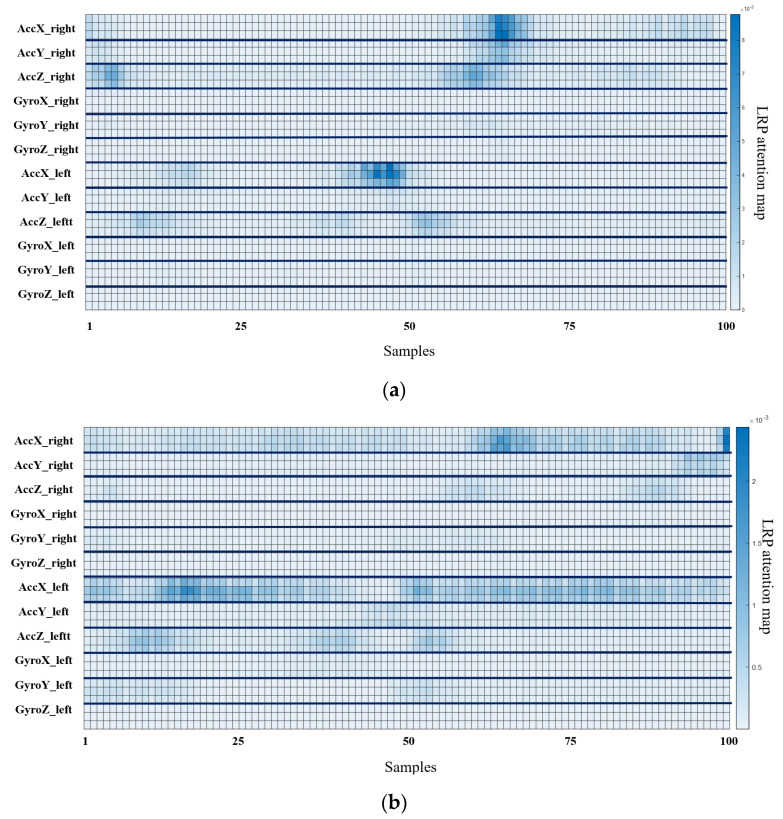
Osteopenia and sarcopenia result of applying LRP to ResNet50. (**a**) LRP result of osteopenia. (**b**) LRP result of sarcopenia.

The output for layer2, when the deep-learning-based XAI technique, LRP, Grad-CAM, and Relevance-CAM were applied to ResNet50, is shown in Figure 7. In ResNet50, the CAM technique shows low resolution in small-sized images because the feature map is reduced in layer2. It is difficult to interpret the CAM results for ResNet50 with input sizes of 100 horizontal and 36 vertical. Therefore, it is desirable to interpret ResNet results as LRP. Figure 8 shows the analysis results of LRP for the ResNet of osteopenia and sarcopenia. The LRP attention map of the osteopenia group had high values in 64~67 samples of the acceleration x-axis of the right foot. Its position is the section where the acceleration value rises after TO, and it is the same as the position in Figure 5a, the parameter-based SHAP result. Osteopenia pays attention to changes in acceleration after TO in SHAP and LRP. The LRP attention map of sarcopenia group has a high value at 99~100 positions of the right acceleration x. The position is the section where HS occurs, and it is the same section as Figure 6a. The result of paying attention to the various sections of the acceleration left is similar to having a high identification result when used as various parameter inputs in SHAP. The sarcopenia group pays attention to the HS section and the various sections of the signal.

## 5. Discussion

The objective of this study was to propose and evaluate a method that can utilize the gait parameters obtained from a wearable device with an inertial sensor in the health management of patients suffering from sarcopenia and osteopenia in daily life.

In the proposed method, the patient was identified using gait phase description-based descriptive statistical parameters as the handcrafted feature-based machine learning input and the original signal of the inertial sensor as the input for the deep learning algorithm. For gait analysis, the identification results were analyzed using XAI tools, such as SHAP and LRP. To verify the proposed gait analysis method, the results of functional tests and questionnaires obtained at the hospital for participants, the results using the existing gait parameters, and the results of the proposed method are discussed. 

To identify osteopenia and sarcopenia, a decrease in walking speed and poor body balance has been reported in previous studies. It has been reported that patients with sarcopenia have a slower walking speed than those with osteopenia. The result of the 3 m TUG was 11.71 s in the sarcopenia group and 10.96 s in the osteopenia group, indicating that the walking speed was slower in the patients with sarcopenia. Except for TUG, statistical significance was not obtained for the MMSE, MFS, SARC-F questionnaire, or BBS. 

In gait analysis using inertial sensors, spatial–temporal parameters have traditionally been used as tools to conveniently identify diseases such as Faller, PD, and THA in everyday life. In this study, to identify patients with osteopenia and sarcopenia, 24 spatial–temporal parameters used for conventional disease identification were detected, and descriptive statistical parameters were detected to analyze the inertial sensor signals according to the gait phase. Statistical significance was obtained for the stance phase, double support phase, and single support phase percent in osteopenia, and SI in the stance and swing phase in sarcopenia. 

With gait analysis using XAI, SHAP demonstrates the importance of parameters and the positive/negative contribution of parameters to the classification results of machine learning. To apply SHAP to machine learning classifiers, it is necessary to obtain a high machine learning accuracy. Good classification results were obtained for osteopenia in XGBoost and sarcopenia in RF. It has been reported that XGBoost has the advantage of being the most accurate among tree-based classifiers, and RF has a strong advantage in terms of overfitting. Comparing various machine learning results, osteopenia showed an accuracy of lower than 70% and sarcopenia showed overfitting. Overfitting in sarcopenia was inferred from the results of the deep learning model. 

From the SHAP results of spatial–temporal parameters, single support phase percent left, double support first phase percent left, double support first phase percent right, double support first phase time left, and stance phase percent right were highly important for the osteopenia group. In the sarcopenia group, double support first phase time left, stance phase percent left, stance phase time SI, double support first phase percent left, and stance phase percent right were found to be of high importance. The important parameters obtained similar results to the statistical analysis; in osteopenia, the phase had a high contribution, whereas in sarcopenia, SI had a high contribution. Double support first phase time left and stance phase percent right showed a high contribution in both groups. The double support first phase time decreased in the osteopenia group compared with that in the sarcopenia group, and the stance phase percentage decreased. An increase in the double support first phase time and an increase in the stance phase percentage indicate a decrease in the walking speed. The time-related parameters were lower in the osteopenia group than in the non-osteopenia group; therefore, it is difficult to identify the osteopenia group based on walking speed. Sarcopenia significantly contributed to the reductions in walking speed and balance parameters, as in the previous study results [1,2,3].

The accuracy of identification of osteopenia patients with spatial–temporal parameters, which is the existing gait analysis parameter, was lower than 70%; thus, it was difficult to analyze the results in SHAP. The inertial sensor had a high temporal resolution; therefore, it was possible to obtain differences between groups by segmenting and analyzing the gait. As a result of SHAP for 840 descriptive statistical parameters, a high contribution from the skewness of the *x*-axis of the accelerometer in the initial swing phase (247) and the absolute sum of values of gyro z in the mid stance (114) were observed for osteopenia. For sarcopenia, the maximum difference between two successive values of accelerometer x in loading response (430) and the absolute sum of values of gyro y in the mid stance (524) showed a high contribution. 

The descriptive statistical parameter 247 for osteopenia was smaller than that for sarcopenia, and the low skewness of osteopenia is due to the rapid occurrence of the maximum value of the acceleration *x*-axis after TO and a large negative value. This result is related to the increase in the swing phase time. The descriptive statistical parameter 430 represents the change in acceleration, and its value of osteopenia is larger than that of the sarcopenia group, implying that the gait speed of the sarcopenia group is slow because the acceleration x is the walking direction. Parameters 114 and 524 are the absolute sum of the gyro values in the mid stance. The absolute sum decreases in osteopenia indicating less foot movement in the mid stance, as shown in Table 14. 

The interpretation of results for LRP-based deep learning was similar to the results of descriptive statistical parameter analysis based on SHAP. However, the reliability was low due to the change in the attention map of the inertial sensor according to the learning results of deep learning as a result of repeated experiments and low identification accuracy. If high-accuracy identification results are obtained, it is expected that the inertial signal characteristics of osteopenia and sarcopenia can be obtained using deep learning.

Functional tests and questionnaires conducted in the hospital were not statistically significant, except for TUG in the sarcopenia group. Spatial–temporal parameters, which have previously been used as gait parameters, showed statistical significance in the sarcopenia group and the osteopenia group, but showed a low identification accuracy of 63% in the osteopenia group. The proposed descriptive statistical parameters obtained an accuracy of 76% or more, and the descriptive statistical parameters attributed similar meanings to the results of the spatial–temporal parameters, had high statistical significance, and can be used as a new clinical tool because the difference in parameter values between the osteopenia and sarcopenia groups is remarkable. Descriptive statistical parameters can be used as useful tools for patient identification and risk detection. 

## 6. Conclusions

The inertial-sensor-based gait signal was acquired and analyzed for patients with osteopenia and sarcopenia. Spatial–temporal parameters used in conventional clinical evaluation and diagnosis are effective tools for understanding gait. However, they have poor temporal resolution and do not include the function of kinematic signals during the gait cycle. Therefore, the inertial sensor data can obtain descriptive statistical parameters for each gait phase. 

For analyzing the patients and control groups, parameters can be statistically analyzed or analyzed through machine-learning-based XAI. To apply XAI, high-accuracy machine learning is required; thus, useful parameters obtained from parameter analysis are used to increase the accuracy of machine learning. Therefore, parameter interpretation is important for patient identification and risk estimation. As a machine learning algorithm, XGBoost for osteopenia and RF for sarcopenia showed high performance, whereas for deep learning, ResNet50, which transfer-learned a human identification model, achieved high accuracy. For the analysis of gait parameters, SHAP was applied to the machine learning model to detect the importance and contribution of the parameters. Unlike Gini and permutation importance, SHAP has advantages of lowering the importance of a parameter when there are similar characteristics between the high-importance parameters. When deep learning identifies patient, the attention map of the inertial sensor signal was analyzed using LRP.

Analyzing the signal of the inertial sensor through XAI, we can diagnose and manage osteopenia and sarcopenia in daily life using a smart insole rather than an expensive clinical tool because the inertial sensor signal contains abundant information on gait. Although the number of participants in this study was extremely small to enable fully understanding the gait characteristics of osteopenia and sarcopenia, the proposed method is effective in analyzing osteopenia and sarcopenia. Therefore, in future studies, additional clinical evaluations will be performed to obtain and analyze many patients and segment data according to sex, age, and dominant leg.

## Figures and Tables

**Table 1:** Existing studies on disease identification using gait parameters. Abbreviations are as shown in Table A1.

Reference	Parameter	Disease	Position	Classification	Accuracy
Caramia 2018 [9]	Step length, step time, stride time, speed, hip, knee, and ankle ROM	PD	R and L ankle, knee, hip, chest	LDA, NB, k-NN, SVM, SVM RBF, DT, majority of votes	96.88%
Eskofier 2016 [10]	Energy maximum, minimum, mean, variance, skewness, kurtosis, fast Fourier transform	PD	Upper limbs	AdaBoost, PART, k-NN, SVM, CNN	90.9%
Howcroft 2017 [11]	Cadence, stride time maximum, mean, and SD of acceleration	Faller	Head, pelvis, R and L shank	NB, SVM, NN	57%
Tunca 2019 [12]	Stride length, cycle time, stance time, swing time, clearance, stance ratio, cadence, speed	Faller	Both feet	SVM, RF, MLP, HMM, LSTM	94.30%
Teufl 2019 [5]	Stride length, stride time, cadence, speed, hip and pelvis ROM	THA	Hip, thigh, shank, foot	SVM	97%
Dindorf 2020 [13]	Various parameters	THA	Hip, knee, pelvis, ankle	RF, SVM, SVM RBF, MLP	100%
Kim 2021 [4]	Various parameters	Sarcopenia	Both feet	RF, SVM, MLP, CNN, BiLSTM	95%
Ours	Various parameters	Osteopenia Sarcopenia	Both feet	RF, SVM, XGBoost, CNN, BiLSTM, ResNet	88.69% 93.75%

**Table 2:** Group population statistics for osteopenia and sarcopenia groups.

Parameter	Osteopenia	Non-Osteopenia	Osteopenia *p*-Value	Sarcopenia	Non-Sarcopenia	Sarcopenia *p*-Value
Age (years)	70.48 ± 2.36	70.33 ± 2.56	0.852	71.10 ± 2.13	69.50 ± 3.14	0.199
Height (cm)	153.65 ± 4.83	152.80 ± 5.93	0.614	150.87 ± 4.66	153.10 ± 4.36	0.283
Weight (kg)	57.75 ± 6.12	59.57 ± 7.12	0.379	53.55 ± 5.62	61.20 ± 5.07	0.005
Feet_size (mm)	236.91 ± 7.66	238.57 ± 6.55	0.453	232.00 ± 5.87	239.50 ± 6.43	0.014
MMSE	27.62 ± 1.77	28.19 ± 1.78	0.303	27.80 ± 1.40	27.30 ± 2.16	0.547
SARC-F	3.19 ± 2.40	3.86 ± 2.15	0.349	2.90 ± 1.52	2.90 ± 2.85	1.000
MFS	23.10±17.92	26.43 ± 16.59	0.535	13.50 ± 12.92	23.50 ± 12.70	0.098
BBS	42.38 ± 8.48	42.19 ± 6.85	0.937	43.10 ± 6.26	41.90 ± 9.47	0.742
3m TUG	10.96 ± 1.64	11.50 ± 2.87	0.464	11.71 ± 1.62	9.85 ± 1.92	0.031
Grasp_right (kg)	17.29 ± 5.42	18.77 ± 4.71	0.351	14.42 ± 3.65	22.57 ± 2.73	0.000
Grasp_left (kg)	17.61 ± 4.67	18.04 ± 4.40	0.761	14.15 ± 3.97	22.17 ± 3.02	0.000
T_score (DEXA)	−1.85 ± 0.74	0.69 ± 1.49	0.000	−0.49 ± 2.08	−0.64 ± 2.03	0.872
SMI(ASM/height)	5.37 ± 0.55	5.38 ± 0.65	0.961	4.58 ± 0.32	5.93 ± 0.35	0.000

**Table 3:** Definition of gait parameters.

Gait Parameters	Definition
Spatial–temporal parameters	
Cadence	Number of steps acquired per minute
Stance phase (time)	Percent (time) starting with HS and ending with TO of the same foot
Swing phase (time)	Percent (time) starting with TO and ending with HS of the same foot
Single support phase (time)	Percent (time) when only one foot is on the ground
Double support phase (time)	Percent (time) when both feet are on the ground
Stride length	Distance starting with HS and ending with next HS of the same foot
Symmetry indices (SI)	Absolute values of (right—left)/(0.5 × ( right + left )
Descriptive statistical parameters	
Max	Greatest values
Min	Least or smallest values
SD	Standard deviation of values
AbSum	Absolute sum of values
Root-mean-square (RMS)	Arithmetic mean of the squares of a set of values
Kurtosis	Assesses whether the tails of a given distribution contain extreme values
Skewness	A measure of the asymmetry of the probability distribution of a real-valued random variable about its mean
MMgr	Gradient from maximum value to minimum value
DMM	Difference between maximum value and minimum value
Mdif	Maximum for the difference between two successive values

**Table 4:** Instantiation of deep learning model.

CNN		BiLSTM		ResNet50	
Input	None, 100, 36, 1	Input	None, 100, 36, 1	Input	None, 100, 36, 1
Conv1	3×3, 5 2×1 max pooling,	BiLSTM1	5	Conv1	7×7, 64 stride 2 3×3 max pooling, stride 2
Conv2	3×3, 5 2×1 max pooling,	BiLSTM2	10	Conv2	$\left[\begin{array}{c} 1\times 1,\;64\\ 3\times 3,\;64\\ 1\times 1,\;256 \end{array}\right]\times 3$
Conv3	3×3, 20	Dropout	0.5	Conv3	$\left[\begin{array}{c} 1\times 1,\;128\\ 3\times 3,\;128\\ 1\times 1,\;512 \end{array}\right]\times 4$
Dropout	0.5		FC, Dense	Conv4	$\left[\begin{array}{c} 1\times 1,\;256\\ 3\times 3,\;256\\ 1\times 1,\;1024 \end{array}\right]\times 6$
	FC, Dense			Conv5	$\left[\begin{array}{c} 1\times 1,\;512\\ 3\times 3,\;512\\ 1\times 1,\;2048 \end{array}\right]\times 3$
					GAP, FC

**Table 5:** Identification result of RF, XGBoost, and SVM (accuracy, precision, recall and F1-score).

Groups	Parameters	Models	Accuracy	Precision	Recall	F1-Score
Osteopenia	Spatial–temporal (24)	RF	0.494	0.476	0.370	0.393
		XGBoost	0.476	0.476	0.376	0.406
		SVM	0.637	0.619	0.511	0.544
	Descriptive statistical (100)	RF	0.649	0.655	0.612	0.607
		XGBoost	0.684	0.690	0.680	0.650
		SVM	0.607	0.678	0.590	0.604
Sarcopenia	Spatial–temporal (24)	RF	0.802	0.825	0.775	0.775
		XGBoost	0.752	0.725	0.667	0.677
		SVM	0.775	0.603	0.775	0.658
	Descriptive statistical (100)	RF	0.675	0.675	0.632	0.631
		XGBoost	0.603	0.675	0.557	0.591
		SVM	0.637	0.704	0.657	0.644

**Table 6:** Identification result of CNN, BiLSTM, and ResNet (accuracy, precision, recall and F1-score).

Groups	Models	Accuracy	Precision	Recall	F1-Score
Osteopenia	CNN	0.696	0.690	0.735	0.670
	BiLSTM	0.619	0.570	0.610	0.571
	ResNet	0.767	0.672	0.726	0.676
	ResNet(transfer)	0.786	0.869	0.747	0.787
Sarcopenia	CNN	0.600	0.437	0.525	0.447
	BiLSTM	0.425	0.300	0.350	0.299
	ResNet	0.612	0.337	0.500	0.394
	ResNet(transfer)	0.700	0.612	0.636	0.606

**Table 7:** Osteopenia identification results according to the number of important parameters (accuracy, %).

Class	ML	Number of Parameters										
		2	3	4	5	6	7	8	9	10	20	100
Gini	RF	70.83	70.23	64.88	72.02	68.45	63.69	61.30	60.11	60.71	61.30	64.88
	XGBoost	66.66	67.85	64.88	71.42	68.45	64.28	65.47	61.30	65.47	67.26	68.45
	SVM	64.28	64.88	64.88	64.28	61.30	61.30	59.52	55.35	57.73	58.33	60.71
Permutation	RF	73.21	70.83	69.64	67.26	64.28	68.45	70.23	69.04	67.26	67.26	64.88
	XGBoost	69.64	70.83	70.23	68.42	64.88	65.47	67.26	66.70	67.26	70.23	68.45
	SVM	65.47	68.45	66.07	64.28	66.66	66.66	64.28	64.88	64.88	60.71	60.71
SHAP	RF	73.80	76.19	70.23	63.69	63.09	63.69	63.09	63.69	57.73	60.11	64.88
	XGBoost	70.23	75	74.40	73.21	66.66	67.85	63.69	59.52	56.54	68.45	68.45
	SVM	71.42	71.42	67.26	61.30	58.33	58.33	57.14	55.95	57.14	62.5	60.71

**Table 8:** Sarcopenia identification results according to the number of important parameters (accuracy, %).

Class	ML	Number of Parameters										
		2	3	4	5	10	15	16	17	18	20	100
Gini	RF	50	58.75	62.5	65	68.75	67.5	68.75	71.25	71.25	62.5	67.5
	XGBoost	52.5	57.5	65	66.25	62.5	58.75	58.75	58.75	58.75	58.75	60
	SVM	52.5	58.75	66.25	65	72.5	57.5	56.25	56.25	58.75	60	63.75
Permutation	RF	62.5	60	56.25	53.75	57.5	67.5	55	60	70	62.5	67.5
	XGBoost	60	60	55	58.75	65	63.75	68.75	65	66.25	67.5	60
	SVM	61.25	60	60	55	65	66.25	66.25	68.75	63.75	60	63.75
SHAP	RF	56.25	60	57.5	65	67.5	62.5	72.5	73.75	68.75	67.5	67.5
	XGBoost	46.25	63.75	62.5	65	65	63.75	63.75	65	66.25	63.75	60
	SVM	58.75	67.5	60	61.25	675	66.25	68.75	62.5	60	58.75	63.75

**Table 9:** Feature importance and Shapley values of descriptive statistical parameters.

**Class**	**Important Parameter**	**1**	**2**	**3**	**4**	**5**	**6**	**7**	**8**	**9**	**10**
Osteopenia	Parameters	247	114	87	218	816	206	291	21	169	667
	Shapley value	0.97	0.28	0.27	0.2	0.18	0.17	0.16	0.13	0.1	0.09
Sarcopenia	Parameters	430	524	51	9	270	457	231	387	3	97
	Shapley value	0.66	0.28	0.25	0.22	0.17	0.16	0.15	0.13	0.13	0.13
**Class**	**Important Parameter**	**11**	**12**	**13**	**14**	**15**	**16**	**17**	**18**	**19**	**20**
Osteopenia	Parameters	774	117	45	802	312	23	542	242	554	422
	Shapley value	0.09	0.08	0.08	0.07	0.07	0.07	0.07	0.06	0.06	0.06
Sarcopenia	Parameters	5	67	521	690	607	704	380	469	8	257
	Shapley value	0.13	0.12	0.11	0.09	0.09	0.08	0.08	0.08	0.08	0.07

**Table 10:** Seven-phase descriptive statistical parameters.

		Right										Left									
	Parameter	Max	Min	SD	AbSum	RMS	Ku	Ske	MMgr	DMM	Mdif	Max	Min	SD	AbSum	RMS	Ku	Ske	MMgr	DMM	Mdif
Loading response	AccX	1	2	3	4	5	6	7	8	9	10	421	422	423	424	425	426	427	428	429	430
	AccY	11	12	13	14	15	16	17	18	19	20	431	432	433	434	435	436	437	438	439	440
	AccZ	21	22	23	24	25	26	27	28	29	30	441	442	443	444	445	446	447	448	449	450
	GyroX	31	32	33	34	35	36	37	38	39	40	451	452	453	454	455	456	457	458	459	460
	GyroY	41	42	43	44	45	46	47	48	49	50	461	462	463	464	465	466	467	468	469	470
	GyroZ	51	52	53	54	55	56	57	58	59	60	471	472	473	474	475	476	477	478	479	480
Mid stance	AccX	61	62	63	64	65	66	67	68	69	70	481	482	483	484	485	486	487	488	489	490
	AccY	71	72	73	74	75	76	77	78	79	80	491	492	493	494	495	496	497	498	499	500
	AccZ	81	82	83	84	85	86	87	88	89	90	501	502	503	504	505	506	507	508	509	510
	GyroX	91	92	93	94	95	96	97	98	99	100	511	512	513	514	515	516	517	518	519	520
	GyroY	101	102	103	104	105	106	107	108	109	110	521	522	523	524	525	526	527	528	529	530
	GyroZ	111	112	113	114	115	116	117	118	119	120	531	532	533	534	535	536	537	538	539	540
Terminal stance	AccX	121	122	123	124	125	126	127	128	129	130	541	542	543	544	545	546	547	548	549	550
	AccY	131	132	133	134	135	136	137	138	139	140	551	552	553	554	555	556	557	558	559	560
	AccZ	141	142	143	144	145	146	147	148	149	150	561	562	563	564	565	566	567	568	569	570
	GyroX	151	152	153	154	155	156	157	158	159	160	571	572	573	574	575	576	577	578	579	580
	GyroY	161	162	163	164	165	166	167	168	169	170	581	582	583	584	585	586	587	588	589	590
	GyroZ	171	172	173	174	175	176	177	178	179	180	591	592	593	594	595	596	597	598	599	600
Pre swing	AccX	181	182	183	184	185	186	187	188	189	190	601	602	603	604	605	606	607	608	609	610
	AccY	191	192	193	194	195	196	197	198	199	200	611	612	613	614	615	616	617	618	619	620
	AccZ	201	202	203	204	205	206	207	208	209	210	621	622	623	624	625	626	627	628	629	630
	GyroX	211	212	213	214	215	216	217	218	219	220	631	632	633	634	635	636	637	638	639	640
	GyroY	221	222	223	224	225	226	227	228	229	230	641	642	643	644	645	646	647	648	649	650
	GyroZ	231	232	233	234	235	236	237	238	239	240	651	652	653	654	655	656	657	658	659	660
Initial swing	AccX	241	242	243	244	245	246	247	248	249	250	661	662	663	664	665	666	667	668	669	670
	AccY	251	252	253	254	255	256	257	258	259	260	671	672	673	674	675	676	677	678	679	680
	AccZ	261	262	263	264	265	266	267	268	269	270	681	682	683	684	685	686	687	688	689	690
	GyroX	271	272	273	274	275	276	277	278	279	280	691	692	693	694	695	696	697	698	699	700
	GyroY	281	282	283	284	285	286	287	288	289	290	701	702	703	704	705	706	707	708	709	710
	GyroZ	291	292	293	294	295	296	297	298	299	300	711	712	713	714	715	716	717	718	719	720
Mid swing	AccX	301	30	303	304	305	306	307	308	309	310	721	722	723	724	725	726	727	728	729	730
	AccY	311	312	313	314	315	316	317	318	319	320	731	732	733	734	735	736	737	738	739	740
	AccZ	321	322	323	324	325	326	327	328	329	330	741	742	743	744	745	746	747	748	749	750
	GyroX	331	332	333	334	335	336	337	338	339	340	751	752	753	754	755	756	757	758	759	760
	GyroY	341	342	343	344	345	346	347	348	349	350	761	762	763	764	765	766	767	768	769	770
	GyroZ	351	352	353	354	355	356	357	358	359	360	771	772	773	774	775	776	777	778	779	780
Terminal swing	AccX	361	362	363	364	365	366	367	368	369	370	781	782	783	784	785	786	787	788	789	790
	AccY	371	372	373	374	375	376	377	378	379	380	791	792	793	794	795	796	797	798	799	800
	AccZ	381	382	383	384	385	386	387	388	389	390	801	802	803	804	805	806	807	808	809	810
	GyroX	391	392	393	394	395	396	397	398	399	400	811	812	813	814	815	816	817	818	819	820
	GyroY	401	402	403	404	405	406	407	408	409	410	821	822	823	824	825	826	827	828	829	830
	GyroZ	411	412	413	414	415	416	417	418	419	420	831	832	833	834	835	836	837	838	839	840

**Table 11:** Osteopenia and sarcopenia identification results with the 20 parameters from Table 9 (accuracy, %).

**Class**	**Important Parameter**	**1**	**2**	**3**	**4**	**5**	**6**	**7**	**8**	**9**	**10**
Osteopenia	RF	x	75	85.11	85.71	78.57	82.14	80.95	81.54	77.97	76.78
	XGBoost	x	72.02	80.95	88.69	87.69	87.5	85.11	82.73	81.54	83.33
	SVM	x	74.40	75	75.59	83.92	82.73	80.95	81.54	80.35	78.57
Sarcopenia	RF	x	85	82.5	83.75	85	85	86.25	82.5	8	82.5
	XGBoost	x	80	72.5	78.75	76.25	73.75	75	71.25	73.75	71.25
	SVM	x	81.25	80	82.5	81.25	82.5	86.25	86.25	87.5	81.25
**Class**	**Important Parameter**	**11**	**12**	**13**	**14**	**15**	**16**	**17**	**18**	**19**	**20**
Osteopenia	RF	77.38	72.61	78.57	74.40	79.16	82.14	79.76	73.80	82.14	82.73
	XGBoost	76.78	76.78	76.19	77.97	80.95	81.54	77.38	74.40	73.80	74.40
	SVM	76.19	77.97	74.40	72.61	75.59	76.78	76.19	77.97	79.16	74.40
Sarcopenia	RF	81.25	83.75	86.25	88.75	86.25	87.5	91.25	93.75	86.25	92.5
	XGBoost	71.52	75	71.25	70	71.25	75.	72.5	72.5	71.25	72.5
	SVM	80	83.75	86.25	83.75	86.25	81.25	83.75	78.75	78.75	78.75

**Table 12:** Spatial–temporal parameters of osteopenia and sarcopenia.

	Parameter	Osteopenia	Non-Osteopenia	Shapley Value	Sarcopenia	Non-Sarcopenia	Shapley Value
1	Stance phase time right (s)	0.61	0.645	0.034 **	0.614	0.608	0.014
2	Stance phase time left (s)	0.612	0.641	0.084 *	0.617	0.604	0.18
3	Swing phase time right (s)	0.427	0.419	0.156	0.416	0.414	0.143
4	Swing phase time left (s)	0.424	0.422	0.04	0.412	0.417	0.039
5	Stance phase percent right (%)	58.77	60.442	0.196 **	59.468	59.445	0.235
6	Stance phase percent left (%)	59.05	60.124	0.035 **	59.853	59.114	0.345
7	Double support first phase time right (s)	0.1	0.115	0.074 **	0.112	0.099	0.005
8	Double support first phase time left (s)	0.085	0.106	0.197 **	0.09	0.090	0.551
9	Double support second phase time right (s)	0.085	0.106	0.031 **	0.09	0.090	0.097
10	Double support second phase time left (s)	0.1	0.115	0.072 **	0.111	0.099	0.007
11	Single support phase time right (s)	0.424	0.422	0.078	0.412	0.418	0.007
12	Single support phase time left (s)	0.427	0.419	0.017	0.416	0.414	0.018
13	Double support first phase percent right (%)	9.66	10.711	0.224	10.802	9.692	0
14	Double support first phase percent left (%)	8.18	9.857	0.311 **	8.563	8.858	0.248
15	Double support second phase percent right (%)	8.17	9.846	0.046 **	8.556	8.855	0.072
16	Double support second phase percent left (%)	9.606	10.686	0.017 **	10.727	9.677	0.001
17	Single support phase percent right (%)	40.939	39.884	0.077 **	40.11	40.897	0.035
18	Single support phase percent left (%)	41.262	39.58	0.416 **	40.562	40.578	0.02
19	Stride length right (m)	0.95	0.93	0.065	0.94	0.979	0.022
20	Stride length left (m)	0.918	0.892	0.015	0.896	0.942	0.011
21	Stance phase time SI	0.031	0.032	0.018	0.036	0.025	0.250 **
22	Swing phase time SI	0.041	0.046	0.073	0.053	0.034	0.049 **
23	Stance phase percent SI	0.026	0.028	0.013	0.0325	0.021	0.007 **
24	Cadence (steps/min)	115.781	113.859	0.047	116.21	117.469	0

**Table 13:** Descriptive statistical parameters of osteopenia and sarcopenia. * indicates that the *p*-value is less than 0.025, and ** indicates that the *p*-value is less than 0.001.

	Osteopenia				Sarcopenia			
	Parameter	Osteopenia	Non-Osteopenia	Shapley Value	Parameter	Sarcopenia	Non-Sarcopenia	Shapley Value
1	247	0.126	0.548	1.033 **	430	2.748	3.797	0.921 **
2	114	1.892	2.613	0.312 **	524	4.925	2.403	0.113 **
3	87	0.357	1.201	0.247 **	51	0.813	0.463	0.189 **
4	218	5.671	7.065	0.200 **	9	8.121	11.813	0.142 **
5	816	3.091	2.502	0.055 **	270	16.417	13.079	0.304 **
6	206	1.926	2.089	0.119 *	457	−0.352	0.047	0.003 **
7	291	3.774	3.129	0.020 **	231	1.532	0.891	0.002 **
8	21	35.175	29.313	0.023 **	387	−0.17	0.042	0.002 **
9	169	3.563	2.823	0.032 **	3	2.267	3.44	0.129 **
10	667	0.135	0.481	0.153 **	97	−0.425	0.274	0.021 **

**Table 14:** Top 2 descriptive statistical parameters of osteopenia and sarcopenia.

Parameter	Osteopenia	Non-Osteopenia	Sarcopenia	Non-Sarcopenia
247	0.126 + 0.425	0.548 + 0.382	0.364 + 0.483	0.327 + 0.534
114	1.892 + 0.86	2.613 + 0.938	2.078 + 1.088	2.217 + 0.591
430	3.292 + 1.05	3.285 + 0.818	2.748 + 0.833	3.797 + 0.813
524	3.317 + 2.098	4.297 + 4.873	4.925 + 3.479	2.403 + 0.473

## Data Availability

The data are not publicly available due to company security policy and personal protection of subjects. Data are available from the authors upon reasonable request.

## Appendix A

**Table A1:** Abbreviations.

Abbreviations	Raw	Abbreviations	Raw
XAI	eXplainable Artificial Intelligence	LDA	Linear Discriminant Analysis
BMD	Bone Mineral Density	NB	Naïve Bayes
SD	Standard Deviation	k-NN	k-Nearest Neighbor
DEXA	Dual-Energy X-ray Absorptiometry	SVM	Support Vector Machines
PD	Parkinson’s Diseases	RBF	Radial Basis Function
THA	Total Hip Arthroplasty	DT	Decision Tree
IMU	Inertial Measurement Unit	XGBoost	Extreme Gradient Boosting
HS	Heel Strike	HMM	Hidden Markov Model
TO	Toe Off	RF	Random Forest
LIME	Local Interpretable Model-agnostic Explanations	ANN	Artificial Neural Network
SHAP	SHapley Additive exPlanations	CNN	Convolutional Neural Network
SMI	Skeletal Muscle mass Index	LSTM	Long Short-Term Memory
MMSE	Mini-Mental State Examination	ResNet	Residual neural Network
MFS	Mores Fall Scale	GAP	Global Average Pooling
TUG	Timed Up and Go	FC	Fully Connected
BBS	Berg Balance Scale	LRP	Layer-wise Relevance Propagation
ROM	Range of Motion	CAM	Class Activation Mapping
